# Transposable elements promote the evolution of genome streamlining

**DOI:** 10.1098/rstb.2020.0477

**Published:** 2022-01-17

**Authors:** Bram van Dijk, Frederic Bertels, Lianne Stolk, Nobuto Takeuchi, Paul B. Rainey

**Affiliations:** ^1^ Department of Microbial Population Biology, Max Planck Institute for Evolutionary Biology, Plön, Germany; ^2^ Theoretical Biology, Department of Biology, Utrecht University, The Netherlands; ^3^ School of Biological Sciences, University of Auckland, Auckland, New Zealand; ^4^ Laboratory of Biophysics and Evolution, CBI, ESPCI Paris, Université PSL, CNRS, Paris, France

**Keywords:** lineage selection, non-transitive interactions, sex, horizontal gene transfer, spatial structure, altruism

## Abstract

Eukaryotes and prokaryotes have distinct genome architectures, with marked differences in genome size, the ratio of coding/non-coding DNA, and the abundance of transposable elements (TEs). As TEs replicate independently of their hosts, the proliferation of TEs is thought to have driven genome expansion in eukaryotes. However, prokaryotes also have TEs in intergenic spaces, so why do prokaryotes have small, streamlined genomes? Using an *in silico* model describing the genomes of single-celled asexual organisms that coevolve with TEs, we show that TEs acquired from the environment by horizontal gene transfer can promote the evolution of genome streamlining. The process depends on local interactions and is underpinned by rock–paper–scissors dynamics in which populations of cells with streamlined genomes beat TEs, which beat non-streamlined genomes, which beat streamlined genomes, in continuous and repeating cycles. Streamlining is maladaptive to individual cells, but improves lineage viability by hindering the proliferation of TEs. Streamlining does not evolve in sexually reproducing populations because recombination partially frees TEs from the deleterious effects they cause.

This article is part of the theme issue ‘The secret lives of microbial mobile genetic elements’.

## 1. Introduction

Prokaryotes and eukaryotes have distinct genome architectures. In general, prokaryotes have small streamlined genomes where up to up to 90% of DNA is host-essential [1,2]. By contrast, eukaryotes have large genomes [3,4] with only a small proportion encoding host-essential proteins [5]. The intergenic space of eukaryotes is populated by numerous repetitive sequences [6–8], many of which are transposable elements (TEs) or remnants thereof. As TEs can replicate independently of hosts, proliferation of TEs is thought to have driven genome expansion in eukaryotes [7,9,10]. Prokaryotes, however, also harbour TEs in intergenic spaces and yet have streamlined genomes [11–15]. If TEs play a role in determining the large genomes of eukaryotes, then why are bacterial genomes more streamlined?

To understand the relationship between TEs and genome architecture, it is necessary to consider mechanisms underpinning TE persistence. Theory predicts that, in asexual organisms, the long-term fate of TEs within a given lineage is extinction [16,17]. Opportunity for TEs to persist in asexual organisms therefore depends critically on ability to periodically invade new lineages via horizontal gene transfer (HGT) [18–20]. In eukaryotic populations, TEs are maintained by sex [21,22]. Although HGT and sex are often assumed to be similar processes, the evolutionary consequences are different. For example, with sex, recombination length scales proportionally to genome size, which may explain why larger genomes favour sex over HGT [23]. Furthermore, HGT often occurs through recombination-independent mechanisms [24–26], with the possibility that integration compromises host integrity [27].

Although TEs can become linked to ecologically relevant genes and thus confer direct benefits to their hosts, the majority incur measurable fitness costs [28,29]. Marginal costs stem from need to replicate the additional DNA that is generated by TE duplication, but more substantive costs arise when TEs integrate into—and therewith inactivate—host-essential genes. Assuming that TEs insert at random, then the risk of gene inactivation is directly related to the proportion of host-essential DNA. In other words, TE infection may be costly for bacteria with streamlined genomes, which have a high proportion of host-essential DNA, and less costly for eukaryotes, which harbour large stretches of non-coding DNA.

Here, we present a coevolutionary model of TEs and their host genomes. The model explicitly considers hosts with a genome that contains stretches of coding DNA and non-coding DNA. Host genomes can be infected by TEs via uptake of extracellular DNA (eDNA). Once infected, TEs replicate within genomes, where integration into essential genes results in cell death and lysis. Lysis liberates TEs back into the eDNA pool. Our model shows that TEs can drive the evolution of genome streamlining in asexual organisms. The process depends on local interactions and is underpinned by rock–paper–scissors dynamics in which populations of cells with streamlined genomes beat TEs, which beat non-streamlined genomes, which beat streamlined genomes, in continuous and repeating cycles. Genome streamlining does not evolve in sexually reproducing populations because recombination partially unlinks TEs from the deleterious effects they cause. Together, our findings provide support for a previously unrecognized role of TEs in the evolution of genome streamlining.

## 2. Results

### (a) An *in silico* model of the coevolution of transposable elements and host genomes

To understand how the interaction between TEs and cells shapes genome architecture, we present an individual-based model of co-evolving TEs and host genomes packaged within cells. We first focus on simple bacteria-like cells, which engage in HGT via environmental pools of DNA, but later extend the model to encompass sexual reproduction. A brief overview of the model is given below. For more insights into the workings of the model, please see §4, the published code (https://github.com/bramvandijk88/selfishDNA) and the explorable model (https://bramvandijk88.github.io/cacatoo/TEs_streamlining/).

**Figure 1.**
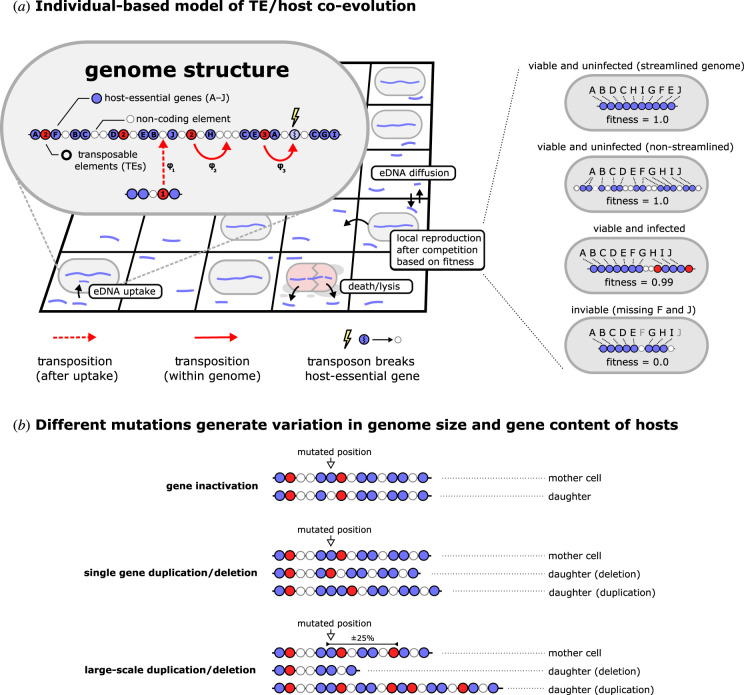
Individual-based model of co-evolving TEs and host genomes. (*a*) Individuals are cells that undergo a process of birth, death and DNA uptake on a spatial grid. Packaged within cells are genomes with three types of genetic elements. A total of 10 host-essential genes (*a*–*j*, in any given order) are necessary for cell viability. There is no explicit cost on the size of the genome, meaning that multiple (redundant) copies of genes may exist, as well as large stretches of non-coding DNA. The genomes also encode TEs that replicate through transposition independent of the host genome. Transposition of TEs happens both after uptake of eDNA (HGT) and during the lifetime of each cell. When transposons insert into coding genes (a host-essential gene or another TE), that gene is inactivated and replaced by a non-coding element. A small fitness cost (*c* = 0.005) is associated with each extra TE copy. The transposition rate of TEs is denoted as *φ*, and may differ among individual TEs. (*b*) Different mutations are depicted for cartoon genomes. Genomes are scanned from left to right upon reproduction, and each position may undergo mutation (illustrated in the cartoon with a white arrow). Mutations generate variation in genome size and gene content of individual cells. Large-scale duplications and deletions affect, on average, 25% of the genome and ensure that genomes do not expand indefinitely (see [30]). As mutations also operate on the level of TEs (i.e., modifying *φ*), the resulting model describes a multi-level coevolutionary process.

Individuals are simple cells that carry a genome with three distinct genetic elements: (i) ten host-essential genes (type A–J), which are necessary for survival/reproduction of the host, (ii) TEs, which are slightly costly to the host and (iii) non-coding DNA, which provides no function, but also carries no cost (figure 1*a*). Elements are represented as a linear sequence and can be exchanged and recombined through different mutational processes (figure 1*b*). For example, single gene duplications may result in redundant gene copies, subsequent gene inactivation may result in the generation of non-coding DNA, and further deletions/duplications may expand or reduce the amount of non-coding DNA.

We assume that a maximally streamlined genome, i.e., a genome encoding only one copy of each essential gene, has the same fitness as a genome that has multiple gene copies and large stretches of non-coding DNA. Fitness differences arise via differences in TE-abundance and site of TE insertion. Insertion of TEs into non-coding DNA or redundant copies of host-essential genes are relatively harmless, incurring a fitness cost of just 0.005 per TE. Insertion into essential genes (lightning symbols in figure 1*a*) is lethal (fitness = 0.0).

At each time step, cells compete locally for space. Space is a limiting resource that becomes available through cell death (see below). When a grid point is empty, competition occurs between up to eight cells from the neighbouring grid points with the winner being chosen at random, but weighted by fitness. The genome of the winning cell is replicated (with mutations) and the daughter cell is placed on the empty grid point.

TEs replicate independently of host genomes. If the rate of transposition (*φ*) is high, host-level selection struggles to prevent accumulation of TEs and ultimately hosts are driven extinct. When *φ* is low, TEs replicate too infrequently to compensate host-level selection and degradation from the eDNA pool. Ultimately, coexistence of TEs and hosts requires the possibility that TEs infect naive (uninfected) lineages. The model assumes that TEs can transpose to the host chromosome after uptake via eDNA. Although such transposition events from naked DNA have been shown to occur [31] by TE-coded determinants [32], they may also arise via transposition from mobile genetic elements such as plasmids or phages [33,34]. The full range of possibilities are encompassed in our model.

After the reproductive phase, non-viable cells plus a small fraction (*d* = 0.02) of the healthy population, die. Dead cells lyse, spilling fragments of genome into the environment giving rise to a pool of eDNA that can be taken up in a transformation-like process by the next generation of cells. Uptake happens at a fixed rate (*u* = 0.01) and integration occurs with the same rate *φ* that determines transposition within genomes. Each time TEs replicate, there is a small chance that the *φ*-parameter changes. Taken together, the model contains multiple levels (TEs within cells within spatially separated populations), with mutation and selection operating on each level. It is important to note that while spatial structure has special relevance to communities forming biofilms [35], spatial structure also manifests at different levels of scale, for example, at the scale of aggregates within soil crumbs [36–38], or root systems of plants. As in our model, the communities in these habitats simultaneously experience different selection pressures owing to the local presence or absence of TEs. Additionally, we make no prior assumptions concerning ecological and evolutionary timescales, except that they overlap sufficiently to yield ecoevolutionary dynamics [39–42].

### (b) Spatial structure allows transposable elements and hosts to coexist

Two important factors affecting the stable maintenance of cells and TEs are the degree of genome streamlining and rate of TE transposition. The manner in which these two properties interact depends on the scale of interactions, and particularly on whether or not interactions are confined to near-neighbours. To explore parameter space, mutation rates were first set to zero, and simulations performed over a range of fixed values of transposition rate (*φ*) and degree of genome streamlining (ratio of host-essential to non-coding DNA). All other parameters are defined in table 1.

**Table 1.** List of model parameters and values (unless stated otherwise). Mutational parameters (marked by an asterisk) are disabled for figure 2.

parameter	value	description
grid size (W, H)	150,150	size of (toroidal) grid on which individuals reside
number of host-essential functions	10	the number of unique functions that a cell must perform to be viable (i.e. the minimal genome size is equal to 10)
fitness penalty per TE (*c*)	0.005	per-TE penalty on fitness
natural death rate (*d*)	0.02	probability of stochastic death for individual cells
DNA uptake (*u*)	0.01	probability of uptake per (local) fragment of DNA (after uptake, the chance of successful integration is determined by the φ-parameter of the TE)
rate of transposition events within genomes (*j*)	0.01	probability of transposition-event during the lifetime of a cell (after this event has been invoked, the chance of successful transposition is further determined by the φ-parameter of the TE)
DNA diffusion (*D*)	0.01	probability of fragments moving into a random neighbouring grid point
DNA degradation (*q*)	0.02	probability of fragments being removed from the grid
TE transposition rate (*φ*)	0.9/evolvable	individual success-rate of transposition (can be unique/evolvable per TE)
TE-induced damage (*b*)	1.0	when a TE inserts into the list of DNA elements (the genome), the adjacent coding genes get inactivated with this probability (otherwise, it is assumed to insert next to it without damaging the gene)
single-gene duplication/deletion*	0.001 per position	per gene per generation probability of duplication or deletion
large scale duplication/deletion/inversion*	0.001 per position	per gene per generation probability of position invoking a large-scale duplication, deletion, or inversion
gene inactivation*	0.001 per position	per gene per generation probability of gene inactivation (position becomes non-coding)
TE transposition mutation*	0.01 per TE	for every TE replication (transposition, host-genome replication), the probability that the transposition rate changes with a uniform step size of ±0.1

**Figure 2.**
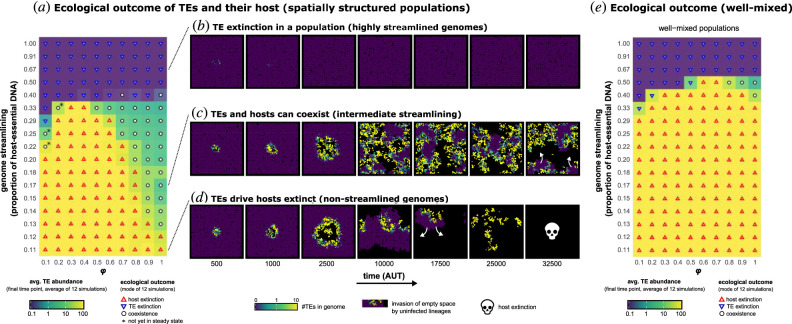
Spatial structure extends opportunity for coexistence of TEs and hosts at intermediate levels of genome streamlining. (*a*) Heatmap shows the results of simulations that explore the relationship between the extent of genome streamlining, rate of transposition (*φ*) and coexistence of TEs and hosts. Simulations marked with an asterisk show apparent coexistence, which after 50 000-time steps have not reached a steady state (see the time courses of TE-abundance for both heatmaps in electronic supplementary material, figure S1). Background colours indicate the average TE abundance in the final time points of the simulations, ranging from zero (purple) to 100 (yellow) on a log scale. (*b*–*d*) Visualizations of TE abundance in spatially structured populations where (*b*) TEs are rapidly lost, (*c*) TEs stably coexist with their host, or (*d*) TEs drive the population to extinction. Colours indicate the number of TEs in genomes, ranging from zero (purple) to one (green) to 10 (yellow). (*e*) Heatmap similar to the one shown in (*a*), but in well-mixed populations. For this, all individuals are assigned a random position on the grid after each round of replication.

As shown in figure 2*a*, spatial structure—and thus local interactions—promotes coexistence of TEs and cells over a range of intermediate levels of genome streamlining and TE transposition rates (*φ*) (white points). TEs are unable to persist in cells that have highly streamlined genomes (high proportion of host-essential DNA; blue inverted triangles), while cells containing less streamlined genomes are susceptible to extinction by TEs (red triangles).

The conditions for coexistence shown in figure 2*a* depend on the interplay between three factors: (i) genome streamlining, (ii) lethal mutations through TE insertion and (iii) the abundance of TEs in the (local) eDNA pool. In cells containing streamlined genomes, infection by TEs is likely to be lethal and thus there is little opportunity for the TEs to increase in frequency (figure 2*b*). At the other extreme, cells with non-streamlined genomes are less likely to be killed by TE infection. This allows TEs to increase in abundance, first within cells, and second in the eDNA pool. The latter then contributes to further amplification of TE abundance via HGT. As the TE load increases there is increasing chance that all cells become infected and from this state, there is no possibility of recovery (figure 2*d*).

Maintenance of uninfected cells (purple space in figure 2*c*) is essential for long-term survival of both TEs and cells. This pool of uninfected cells decreases in frequency when infected by nearby TE-carrying strains, but can increase in frequency by recolonising vacant niche space made available by extinction events (white arrows in figure 2*c*). Uninfected cells are then available for reinfection, resulting in a time-dependent cyclical process with chaotic waves (also see electronic supplementary material, video S1). The critical factor for maintenance of both TEs and hosts is the time-to-extinction (and subsequent cell death and lysis) after infection. If this is too rapid (as happens with highly streamlined genomes), then TEs have little opportunity to amplify within genomes and eventually go extinct. If extinction is too slow, then all cells become infected and all host cells eventually go extinct.

Another important factor determining coexistence is the interaction range. If the infection of one strain readily infects another cell, the uninfected pool of cells is reduced over time. As evident from figure 2*b–d*, spatial structure limits the spread of TEs to the local neighbourhood, which is likely important for coexistence. To test this, the simulations were repeated in well-mixed populations, where individuals are assigned a random position after each round of competition. The results show a highly significant reduction in conditions promoting coexistence (figure 2*e*), thus demonstrating the central importance of spatial structure and local interactions.

### (c) Genome streamlining evolves de novo in a structured environment

An intriguing finding from the above analysis is that streamlined genomes are resistant to invasion by TEs. This is evidently a lineage-level effect. To individual cells with streamlined genomes, infection by a TE is invariably lethal. In other words, what is costly to the individual appears beneficial at the lineage level. A central issue is whether this apparent example of altruism can evolve de novo.

To this end, we introduced TEs into an evolving population of cells containing (initially) non-streamlined genomes. Mutations occur after each replication step, modifying genome size and genome content of hosts, as well as transposition rates of TEs (*φ*) (see §4). Cells in the initial host population are all identical, carrying 10 host-essential genes and 30 non-coding positions, and were locally inoculated with TEs (in the middle of the grid).

**Figure 3.**
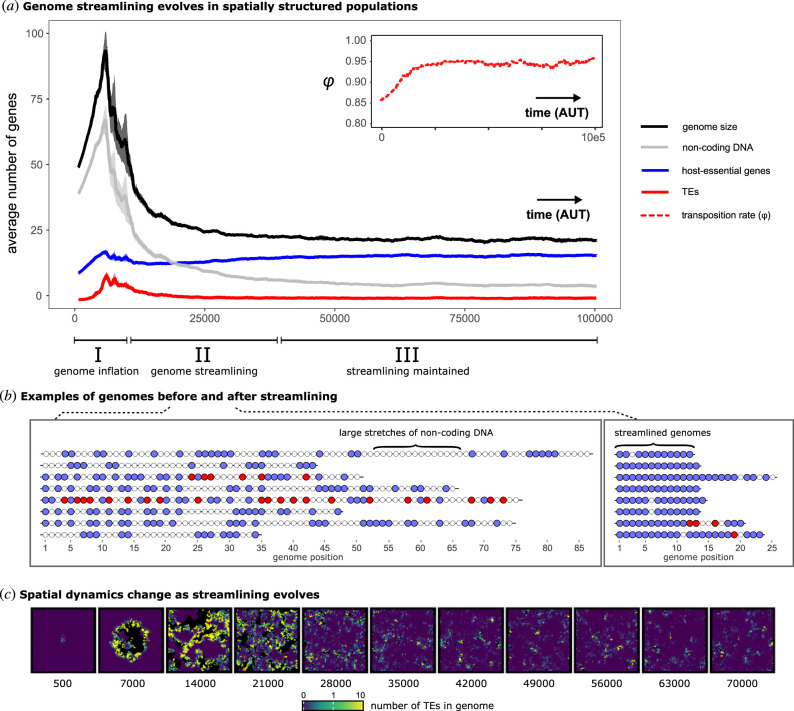
Coevolution of TEs and host genomes drives genome streamlining. (*a*) The average number of genes and genome size are plotted over time. Lines show the average of five independent simulations. Shaded areas denote the standard error across simulations. Host-essential genes are shown in blue, TEs are shown in red, and non-coding positions are shown in grey. Note that TEs persist (i.e. the red line is not zero). The inset shows the evolution of the average transposition rate (*φ*). The ranges of three distinct phases (I, II, and III) in the evolution towards streamlined genomes are shown along the *x*-axis. (*b*) Examples of genomes in populations from (*a*), before and after streamlining has evolved. Blue, red and white dots denote host-essential DNA, TEs and non-coding DNA, respectively. (*c*) TE-abundance per individual, which correspond to equally spaced time points from a single population from (*a*). A log-transformed gradient of blue to green to yellow indicates increasing numbers of TEs inside individual genomes. Black indicates empty space where cells have locally died out, which provides niche space for invasion by other lineages.

Data in figure 3*a* show that host genomes initially expand, but eventually evolve to be more streamlined. Three distinct episodes are notable. Initially (episode I), genomes expand in size. This is a consequence of TE amplification, but also entails an increase in the number of host-essential genes and non-coding elements. After expansion, a period of genome streamlining occurs (episode II). During this phase, a decrease in the number of TEs and non-coding DNA is observed. The decrease in TE-abundance does not reflect a decrease in the transposition rate (*φ*), which instead increases over evolutionary time (figure 3*a*, inset). Thus, TEs do not adapt to their host by becoming less infectious. Eventually, TEs and the amount of non-coding DNA reach a stable equilibrium (episode III), where genomes are comprised primarily of host-essential DNA (figure 3*b*). Concurrent with genome streamlining is a decrease in vacant niche space (black areas in figure 3*c*), which reflects decreased TE-driven extinction events and therewith an increase in the total population size (also see electronic supplementary material, figure S2). In the steady state, most of the population consists of uninfected host cells, with occasional bursts caused by TE infection (figure 3*c*; electronic supplementary material, video S1).

**Figure 4.**
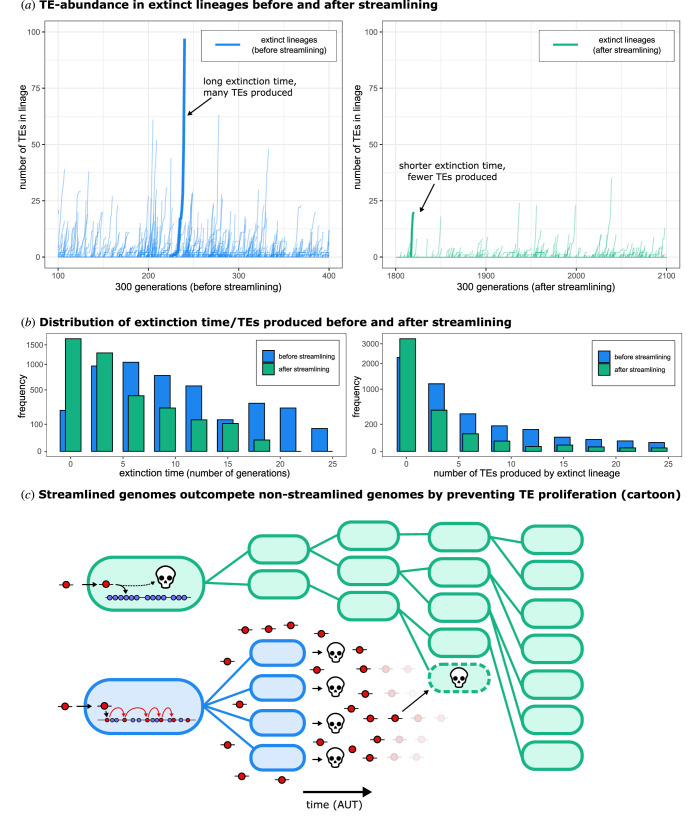
Genome streamlining reduces extinction time and hampers TE proliferation. (*a*) For a single simulation from figure 3, the effects of genome streamlining on lineage viability are illustrated. Before genome streamlining, infected lineages produced more TEs until they went extinct. The *x*-axis shows the generation number, and the *y*-axis the number of TEs (for each extinct lineage during this time interval). The left-hand side shows lineages of cells before streamlining evolved (in blue), and the right-hand side after streamlining has evolved (in green). Two arbitrary lineages are highlighted with a thick line for illustrative purposes. (*b*) For the same simulation as presented in (*a*), histograms are drawn to visualize changes in extinction time (number of generations since infection) and the number of TEs produced by extinct lineages, before and after streamlining. Blue bars are all lineages that went extinct before time point 20 000, whereas green bars are lineages that went extinct between time points 60 000 and 80 000. Each bin represents the total number of occurrences within that time window. Note that the *y*-axes are square-root transformed to clearly illustrate the difference between the two distributions. (*c*) Cartoon illustrating how cells containing streamlined genomes (green), despite spawning fewer progeny in the short term when infected by the same number of TEs, eventually replace cells containing non-streamlined genomes (blue) by limiting opportunities for TE proliferation. Although streamlined genomes may be infected by TEs derived from non-streamlined genomes, newly acquired TEs have little opportunity to amplify because infection of a cell with a streamlined genome is invariably lethal.

As evident in the ecological simulations above (figure 2), the time taken for lineages to go extinct is an important factor and is expected to evolve during the course of the selection experiment. The mechanistic nature of the model means that individual cells can be retrieved after the simulation completes, and their evolutionary history directly observed. Figure 4*a* shows, for each extinct lineage, the number of TEs that accumulate from the time of TE infection until extinction. Lineages of cells with non-streamlined genomes (before streamlining evolved, generations 100–400) persist for longer after infection and liberate many more TEs into the eDNA pool compared to lineages of cells with streamlined genomes (generations 1800–2100). The histograms in figure 4*b* show that streamlined genomes go extinct rapidly (no more than a few generations), and as a consequence only produce a few TEs (figure 4*c*). Thus, although streamlined genomes produce fewer progeny in the short term, they eventually shape an environment in which they thrive. Moreover, after non-streamlined genomes have succumbed to bursts of TE infection, streamlined genomes are free to invade the space freed by cell lysis (figure 4*c*).

### (d) Local interactions are essential for the evolution of genome streamlining

The data shown in figure 3*c* indicate an important role for spatial structure. To test this directly, we repeated the simulations, but assigned individuals to a random position after each round of competition and reproduction. Starting from conditions that are ecologically viable according to figure 2 (10 host-essential genes and 10 non-coding elements), populations rapidly evolved larger genomes and were eventually driven to extinction by TEs (electronic supplementary material, figure S3a). Similar results were obtained when mixing was confined to just the eDNA pool (electronic supplementary material, figure S3b) or when TEs were not amplified by within-cell replication, but exogenously delivered (electronic supplementary material, figure S3c). Moreover, populations that had already evolved genome streamlining (from previous experiments) rapidly went extinct when the local interaction neighbourhood was removed by mixing (electronic supplementary material, figure S2). Local interactions and direct feedback from the environment are thus essential for the evolution and maintenance of streamlined genomes.

### (e) Genome streamlining is driven by transposable element-induced inactivation of host-essential genes

The evolution of genome streamlining appears adaptive in that it results in the elimination of TEs—at least temporarily—from local populations. But to individual cells, genome streamlining is clearly maladaptive: infection of cells with streamlined genomes is invariably lethal. The evolution of genome streamlining thus appears to be attributable to selection at the level of lineage viability, with those lineages comprised of streamlined genomes outcompeting lineages with less streamlined genomes (as illustrated in figure 4*c*).

To test the hypothesis that lineages of cells containing streamlined genomes gain a lineage-level benefit that derives directly from the lethal effects experienced by individual cells, we modified the above simulations. Specifically, we included a parameter *b* that, in the model, scales the likelihood that TEs inactivate a gene at the insertion site. When *b* = 1, TEs always inactivate the (potentially coding) DNA at the insertion site. When *b* = 0, however, TEs are assumed to insert precisely in between two genes, avoiding DNA damage (i.e., TEs have a specific insertion site that does not occur in coding regions). In the latter case, genome streamlining does not evolve (electronic supplementary material, figure S4). However, we found that genome streamlining always evolves when *b* > 0, although genome streamlining evolves very slowly when the risk of DNA damage is low (electronic supplementary material, figure S5).

TEs and their hosts nonetheless persist through continuous waves of infection and recolonization of available niche space. This result demonstrates that the evolution of genome streamlining (figure 3) is driven by TE-generated mutations that are harmful to individual cells.

### (f) Persistence of transposable elements depends on rock–paper–scissors dynamics

Given that cells containing streamlined genomes drive TEs extinct, the persistence of TEs shown in figure 3*c* seems counterintuitive. However, understanding emerges from examination of the eco-evolutionary dynamics (see electronic supplementary material, video S1), combined with observation of the evolution of non-streamlined genomes in the absence of TEs. Starting with the latter, data in electronic supplementary material, figure S6 show that non-streamlined genomes replace streamlined genomes in the absence of TEs. This occurs in part as a consequence of duplication bias (in a minimal genome, deletion-mutants are never viable—making duplications the only mutations that change genome size), but also because genome expansion generates multiple copies of essential genes that confer mutational robustness. Thus, in the absence of TEs, selection favours larger genomes.

In data from evolutionary simulations (figure 3), TEs are never absent (electronic supplementary material, figure S7). Instead, they decline to low numbers in local patches and once rare, individual cells with non-streamlined genomes are favoured over cells with streamlined genomes. Apart from the selective benefits of larger genomes described above, this also occurs because non-streamlined genomes are, at least initially, less sensitive to the deleterious effects of TE infection. However, as the load of TEs within lineages increases, costs are increasingly realized at the level of local lineages. This then establishes conditions that once again favour the evolution of cells with streamlined genomes.

**Figure 5.**
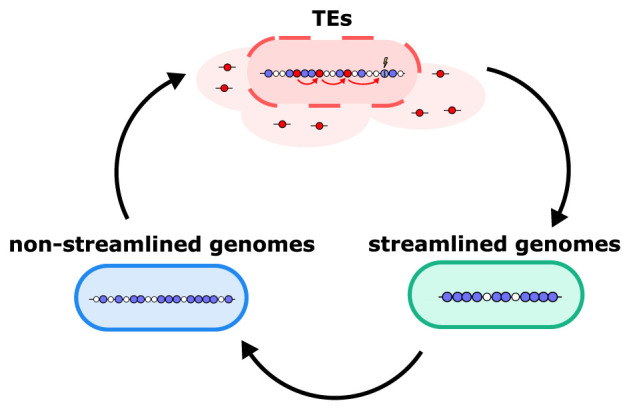
Rock–paper–scissors dynamics allows TEs and cells with streamlined genomes to coexist. A cartoon illustrating how both streamlined genomes and TEs can be maintained within the population indefinitely. As streamlining lowers the (local) abundance of TEs, non-streamlined genomes are favoured. This enables TEs to once again infect cells and locally thrive, which in turn upholds the selection pressure for streamlined genomes.

Cells with streamlined genomes thus beat TEs, which beat non-streamlined genomes, which beat streamlined genomes, and so on, in a cyclical game of rock–paper–scissors (figure 5). The long-term persistence of TEs and cells with streamlined genomes depends on this dynamic. As the re-emergence of non-streamlined genomes entails evolution, disabling mutation in populations that evolved streamlined genomes breaks the rock–paper–scissors cycle, eventually driving TEs extinct (electronic supplementary material, figure S8).

### (g) Transposable elements do not drive genome streamlining in sexually reproducing populations

**Figure 6.**
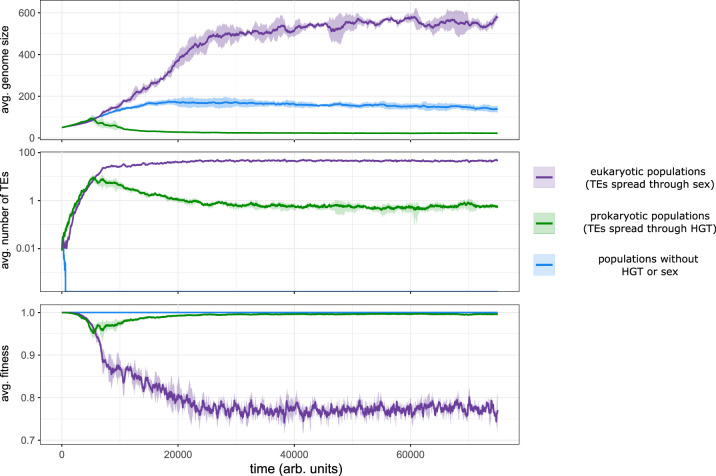
Streamlining does not evolve in sexually reproducing populations because recombination partially unlinks TEs from the deleterious effects they cause. For three different population types, the genome size, TE-abundance and average fitness are shown over time. Shaded areas are the standard errors from five independent simulations. Eukaryotic (sexual) populations are shown in purple, prokaryotic (asexual) populations are shown in green, and populations without HGT or sex are shown in blue.

As TEs require transfer to new linages to persist, simulations in which DNA uptake is disabled result in TE extinction (electronic supplementary material, figure S6). However, TEs in nature can also persist in populations through sex and recombination. Our model was therefore modified to incorporate sexual reproduction by disabling DNA uptake and implementing a simplified form of sexual reproduction. In these populations, competition for vacant space is determined by sampling two individuals from the local neighbourhood (weighed by their fitness), and their genomes recombined via a single cross-over event (see §4). Importantly, this process allows TEs to infect new lineages without transposition, removing the risk of lethal mutations. These sexually reproducing ‘eukaryotic' populations (figure 6, purple lines) did not evolve genome streamlining and grew large in size compared to prokaryotic populations (figure 6, green lines). Accordingly, the average fitness of sexual populations is relatively low, as a substantial fraction of the population was infected with a large number of TEs. In the absence of sex and HGT (i.e., in strictly clonal populations) TEs went extinct and genome size increased (figure 6, blue lines).

To understand the lack of genome streamlining in sexual populations, it is necessary to reconsider the two steps of successful TE amplification. First, a TE must infect a host, but in order to increase in frequency, it must also replicate within the host (at least once). In asexual populations, cells with streamlined genomes die during the infection step, thus immediately blocking further TE amplification. With recombination, however, TEs can infect new lineages without risk of immediately killing the host, irrespective of the level of genome streamlining. Although subsequent transposition may still render the host inviable, the host cannot prevent a TE from infecting its genome, thus allowing the TE to replicate at least once. The fact that genome streamlining does not occur in these sexual populations suggests that streamlining in asexual populations evolves to prevent transposition *between* genomes, and not transposition *within* genomes.

## 3. Discussion

Here, we have presented an *in silico* coevolutionary model of TEs and host genomes. The model reveals an interesting interplay between genome streamlining (the amount of coding DNA) and TE-abundance. Selection initially favours cells with expanded genomes, because additional genome space reduces the chance that transposition has deleterious effects. However, while adaptive at the level of individual cells, cells with expanded genomes provide opportunity for the population of TEs to increase in the lineage of descendent cells to the point where extinction of the lineage becomes inevitable. When the environment is spatially structured, such extinction events are localized, enabling the more persistent (streamlined) genomes to recolonize vacant niche space. We found that the resulting coexistence of TEs and hosts is remarkably stable, which can be explained by rock–paper–scissors interactions [39,43,44], similar to those observed in susceptible–infectious–recovered (SIR) models [45]. Finally, in sexually reproducing populations, streamlined genomes have no advantage over non-streamlined genomes because recombination unlinks TE-infection from potential DNA damage. Thus, our coevolutionary model of TEs and hosts provides an explanation for streamlined genomes in prokaryotes, and expanded genomes in eukaryotes.

Interestingly, genome streamlining is maladaptive at the individual cell level, but is selectively favoured because of benefits that accrue to lineages of cells. This is analogous to abortive infection, a well-studied mechanism that protects cellular collectives against bacteriophages [46]. Earlier modelling on spatially structured populations has already illustrated that early death can be favoured when it promotes the long-term survival of the lineage [47,48]. Our results connect these observations to the evolution of genome architecture, showing that early death is an evolutionarily attainable (and maintainable) protection mechanism against TEs.

The TEs in our model are based on insertion sequence (IS) elements, a particular yet common class of TEs. TEs are assumed to be autonomous (they encode their own transposase function), show no notable bias in insertion site preference, and move both vertically and horizontally. Not all TEs are marked by these characteristics. For example, REPIN sequences (repetitive extragenic palindromic sequences forming a hairpin) take up many intergenic spaces in *E. coli* and *Pseudomonas fluorescens* SBW25 [15,17]. However, REPIN sequences do not move autonomously and are replicated by a single-copy transposase that has been vertically inherited for millions of years [15]. The pattern of REPIN sequence abundance may therefore be explained by a direct fitness advantage that would evidently not promote genome streamlining.

Questions unanswered through use of *in silico* models concern relevance to the natural world. However, there are reasons to assume the likelihood of legitimate connections, particularly given the spatially structured nature of microbial populations, the abundance of TEs and pervasiveness of HGT. Evidence could be sought by interrogation of genome sequences from a set of phylogenetically related strains sampled at precise spatial and temporal scales. Such a future project would be a major challenge, however, as our model abstracts away from the precise magnitude of those spatial and temporal scales. For example, although the ecological processes illustrated in our study are reminiscent of biofilms, the importance of local extinctions and recolonization is perhaps better captured by processes on a larger scale, such as microbial populations growing on segregated food particles with limited exchange. An alternative possibility is to compare data on the relationship between genome size and TE-abundance derived from the analysis of diverse genome sequences with theoretical predictions of this relationship at equilibrium. The latter can be derived from our model populations by analysis of the genomes of all viable cells present at the end of the simulations. The data, shown in electronic supplementary material, figure S7, indicate a strong positive correlation between genome size and TE-abundance only under conditions where TEs cause harmful effects. Precisely such a relationship has been previously reported [49] for IS-elements, with the authors suggesting that such a relationship might indeed reflect robustness of larger genomes to lethal TE insertion. Finally, one could investigate genomes that appear to be outliers, such as asexually reproducing bdelloid rotifers. Despite the absence of sexual reproduction, TEs are persistent in rotifer genomes, which seemingly disagrees with theory. However, multiple studies have now shown that HGT is prevalent in rotifers [19,50], which in accordance with our model could explain why rotifers have a relatively high gene density compared to other eukaryotes [51].

For illustrative purposes, we have deliberately not included other mechanisms that are known to result in genome streamlining. For example, alternative hypotheses for the different structures of prokaryotic and eukaryotic genomes are differential energy budgets [52], deletion biases in prokaryotes [53,54], and the small population sizes of eukaryotes [55]. For prokaryotes, in particular, recent studies have suggested that natural transformation may play an important role in the removal (rather than the acquisition) of mobile elements [56,57]. Furthermore, it is possible that the mechanisms of genetic exchange (sex and/or HGT) are themselves under selection for other reasons, such as the optimal recombination length [23]. Preliminary results for example indicate that a strong deletion bias promotes genome streamlining irrespective of the dynamics of transposons, but TE-induced DNA damage still has a major impact on genome dynamics (electronic supplementary material, figure S9). Thus, although we illustrate that our mechanism can operate in isolation, it is likely that it interplays with a range of additional factors. Clearly, disentangling the many selection pressures that operate on cells and mobile DNA remains a major challenge for future modelling and comparative genomics.

## 4. Methods

The model implemented in this study is an individual-based model (IBM) of the coevolution of TEs and their host. The primary goal of the model is to first explore the conditions under which autonomously replicating TEs can coexist with their host, and how the insertion of TEs into coding DNA shapes the composition of the host genome.

The model is composed of a (toroidal) spatial grid, which is a computationally efficient way of modelling local interactions. On this grid, three types of entities can reside: (i) simple cells with genomes packaged within them, (ii) naked DNA from prior generations that is taken up by cells, and (iii) TEs that can replicate within genomes and transfer horizontally via the eDNA pool (figure 1 in main text). For the precise order in which these entities are updated, see electronic supplementary material methods. It is assumed that TEs are simple selfish genetic elements that replicate/integrate with rate *φ* (see §4d), and serve no purpose to their host. Besides TEs, simple genomes contain host-essential genes and non-coding DNA (see §4a). The exact proportion of non-coding to coding DNA may vary through mutations (see §4b) that occur when cells replicate after local competition has occurred (see §4e). As a consequence of mutation, each TE can also have a unique transposition propensity (*φ*). In short, the model contains multiple levels, describing both the ecology and the evolution of TEs and their host genomes.

### (a) Genome structure and fitness

Individuals carry a genome that encodes a linear sequence of genetic elements. We assume that cells need to perform 10 essential functions, and therewith there exist 10 essential genes (*a*–*j*). We assume that these essential functions are performed when an essential gene with function *i* is present at least once (*e_i_* > 0). Carrying multiple copies of these genes does not directly impact fitness (*f_i_*), which we assume in order to deliberately avoid selecting for streamlining genomes simply due to reduction in costs. However, a genome that lacks one of these essential functions has fitness zero, meaning it cannot (or can no longer) compete for reproduction and dies in the next time step. The second type of genetic elements we consider are TEs, which self-replicate within genomes. The total number of TEs (*T*) confer a small cost (*c*) to the host. The fitness of the host then becomes

$$f_{i}\;=\{\begin{array}{c} 0.0\;\mathrm{if}\;\left(\sum_{n=1}^{k}e_{i}>0\right)\;<\;10\;\left(\mathrm{at}\;\mathrm{least}\;\mathrm{one}\;\mathrm{essential}\;\mathrm{gene}\;\mathrm{missing}\right)\\ 1\;-\;c\;\cdot \;T\;\mathrm{if}\;\left(\sum_{n=1}^{k}e_{i}>0\right)\;\geq \;10(\mathrm{all}\;\mathrm{essential}\;\mathrm{genes}\;\mathrm{present}). \end{array}$$

Note how the third class of genetic elements, non-coding DNA, does not impact fitness. Although it can be generated, amplified, or trimmed through mutational processes, we deliberately avoid implementing costs for genome size to illustrate how TEs drive the streamlining of genomes. Note that our results do not change when including non-essential genes in our fitness function (electronic supplementary material, figure S10).

### (b) Mutational processes

Mutations happen every time genetic elements are replicated, and change the gene content and genome size of individuals. When cells reproduce, their genomes are scanned from left to right, allowing each genetic element to undergo mutations once. Changes can be applied to the genetic element itself, or they can be the start site of a large-scale deletion, duplication, or inversion of multiple genes. These large-scale events enable the rearrangement of gene order, and also ensure that genomes do not grow indefinitely in the absence of a deletion bias [30]. Single genetic elements can be deleted, duplicated or inactivated (transforming host-essential genes and TEs into non-coding genes). TEs can also change their transposition propensity *φ* with a uniform step size (0.1) up or down.

### (c) Death and lysis

Every time step, a small subset of the host population stochastically dies with probability *d*. Moreover, cells that are not viable (or no longer viable due to transposon-induced mutations) also die. Before being removed from the grid, dead cells spill their DNA into the environment. This DNA is uniformly fragmented into pieces of 3 to 8 genetic elements each. These stretches of DNA can either degrade (with rate *q*), or be taken up by future generations (with rate *u*).

### (d) TE-dynamics and HGT

The dynamics of TEs occurs through two distinct processes. The rate at which TEs replicate during the lifetime of a cell is set by parameter *j*. Every time step a cell survives, each TE in its genome gets an opportunity to replicate one with rate *φ*·*j,* where each TE can have a potentially different *φ*-parameter. The second process by which TEs spread is by means of a transformation-like process. Living cells take up naked DNA derived from prior generations with rate *i*, after which TEs can integrate into the host chromosome with the same rate *φ*. We assume that both transposition events (i.e. after uptake or during the cell's lifetime) occur at random positions in the chromosome, and can cause the inactivation of genes at the insertion site (lightning symbols in figure 1*a*). When a gene at the site of transposon-insertion codes for one of the host-essential functions, this event is lethal for cells (unless another copy of that gene is still active). Transposons inserting into non-coding DNA never have a (direct) damaging effect.

In the main text, we also test what happens when inactivation of genes at the insertion site was removed. For these populations, TEs are always inserted ‘next to' rather than ‘into' the genetic element at the insertion site. Note that whether TEs insert ‘into' or ‘next to' genetic elements at the insertion site does not influence their transposition dynamics but only the potential damage that transposition may cause.

Our model assumes that a TE can transpose directly after the uptake of DNA. Although transposition events from naked DNA have been shown to occur [31] by using the machinery encoded on the TE itself [32], our model does not explicitly assume this to be the only mechanism. An alternative route of HGT of a TE would be via an intermediate mobile genetic element such as a plasmid or phage [33,34]. The subsequent transposition to the host chromosome would still carry the risk of transposon-induced mutations. In principle, our model abstracts away from these distinctions.

### (e) Competition and reproduction

Each time step, competition happens for unoccupied grid points. Up to eight cells in the direct (Moore) neighbourhood compete proportional to their fitness. The relative chance that individual *i* wins this competition (*R*_i_) is determined by the individual's fitness (*f*_i_), divided by the total fitness of all competitors (*f*_TOTAL_) plus a constant *ε*. The latter constant ensures that a single individual does not win by default, and ensures that it is unlikely for any individual to reproduce when all competitors are unfit.

$$R_{i}=\frac{\;f_{i}}{\sum_{j}^{n\_\mathrm{neigh}}(\;f_{\mathrm{TOTAL}}){+}_{\;}\varepsilon }$$

### (f) Sexual reproduction

To distinguish the process of HGT from sex and recombination, we implemented a simple mode of sexual reproduction in our model. For this, two competitors are sampled proportional to their fitness (see above), and their genomes are recombined. Because the genes in our model have no sequence identity to infer homology, we cannot model actual homologous recombination, so we instead assume a simple cross-over event that occurs in the middle of the two genomes. The resulting (haploid) genome undergoes mutations in the same way as a clonally reproduced genome would in the base model. These sexually reproducing populations do not take up environmental DNA.

### (g) Parameter choice

Because our model is an abstraction of biological processes, it is not trivial to estimate the precise values that would be realistic/accurate. We however found that, given parameters that allow for host/TE coevolution, our main results are robust to the precise values of parameters. Apart from the parameter sweeps presented throughout this study, we therefore chose to parameterize the model by finding (biologically reasonable) parameter values where:

— **TEs can (potentially) coexist with their host genome**
  • Requires sufficient HGT (or sex) for TEs to jump to new lineages
  • Requires local extinctions such that healthy lineages can invade empty space (through a direct fitness-cost on the TEs, through lethal TE-insertions, or both)
  • DNA diffusion is low, so that TEs can only infect local strains (and not the entire population at once)
— **The model remains computationally feasible**
  • Large-scale duplications and deletions are assumed, so that genomes do not grow indefinitely [30]
  • The system size is set to the minimal size where local extinctions, wavefronts, and multiple strains can occur simultaneously.

Our evolutionary simulations were performed with the above-mentioned conditions in mind. Other variables (gene content, genome size, transposition rate) are allowed to evolve. See table I for a full list of parameters and their values. Further details on robustness, coexistence conditions and our parameter choice are given in electronic supplementary material (part 3).

### (h) Software used

The individual-based model presented in this study is a C++ extension of Cash (Cellular Automaton simulated hardware), originally written in C by R.J. de Boer and A.D. Staritsk. All analyses were done in R, using the packages ggplot2 [58], dplyr [59].

## Data accessibility

The code for the individual-based model and the scripts to run parameter sweeps are available on Github (https://github.com/bramvandijk88/selfishDNA).

The data are provided in electronic supplementary material [60].
